# *Coleus amboinicus* extract increases transforming growth factor-1β expression in Wistar rats with cisplatin-induced nephropathy

**DOI:** 10.14202/vetworld.2019.1346-1351

**Published:** 2019-08-28

**Authors:** Iwan Sahrial, Rondius Solfaine

**Affiliations:** 1 Department of Basic Veterinary Medicine, Faculty of Veterinary Medicine, Airlangga University Kampus C Unair Surabaya, Indonesia; 2 Department of Pathology and Anatomy, Faculty of Veterinary Medicine, University of Wijaya Kusuma Surabaya, Jl. Dukuh Kupang XXV/54 Surabaya, Indonesia

**Keywords:** cisplatin, *Coleus amboinicus*, nephropathy, transforming growth factor-1β

## Abstract

**Background and Aim:**

*Coleus amboinicus* (CA) plants are known to exert antibacterial and anti-inflammatory effects and demonstrate antiproliferative effects against cancer cells. This study aimed to investigate the activity of CA extract on the expression of transforming growth factor-1β (TGF-1β) in cisplatin-induced nephropathy in Wistar rats (*Rattus norvegicus*).

**Materials and Methods:**

CA was obtained from fresh leaves of CA and was extracted using 96% ethanol maceration. This blinded, controlled, randomized post-test study assigned 24 Wistar rats to three groups (n=8). Negative controls received normal saline (P0), nephropathy was induced in rats by cisplatin (5 mg/kg, IP) (P1), and treated with ethanolic coleus extract (500 mg/kg, PO) (P2), respectively, for 7 days. Nephropathy was induced on the 4^th^ day. All rats were sacrificed on the 8^th^ day for blood and kidney sample collection. Concentrations of blood urea nitrogen (BUN), creatinine, and alkaline phosphatase were analyzed using colorimetric analysis. A semi-quantitative analysis was performed on sectioned kidneys to determine the numbers of positive cells for TGF-1β expression and to evaluate structural and functional alterations in the kidneys using histopathological and immunohistochemical staining.

**Results:**

The concentrations of BUN, creatinine, and alkaline phosphatase from blood samples in the treatment group were significantly lower than those of the control group (p<0.05). Morphological evaluation of the tubular interstitium and glomeruli revealed that necrotic, degenerating, and infiltration of cells significantly decreased in the treatment group compared to the control group (p<0.05). The mean immunostaining scores indicating the presence of TGF-1β were 7.8 in the ethanolic coleus extract group, 3 in the induction group, and 2.3 in the control group. The expression scores for TGF-β1 were significantly different between the ethanolic coleus extract treatment and control group (p<0.05).

**Conclusion:**

Our results suggest that in Wistar rats with cisplatin-induced nephropathy, CA extract inhibits pathological lesions by regulating the renal expression of TGF-1β in areas containing the renal tubules and glomeruli.

## Introduction

Nephropathy is acute kidney failure characterized by varied lesions in glomerulosclerosis, thickening of the glomerular basement membrane, mesangial cell proliferation, decreased glomerular filtration rate, high persistent albuminuria, high arterial blood pressure, and fluid retention. Cisplatin is a drug of choice for treating various types of cancer, including sarcomas and carcinomas [1-3], but is often associated with nephrotoxic side effects. A previous study revealed that a single dose of cisplatin (7 mg/kg) caused nephropathy in 6-7 days and was associated with an increase in serum creatinine, blood urea nitrogen (BUN), and serum albumin levels in rats. The mechanism in cisplatin-induced nephropathy is inhibition of protein synthesis, deoxyribonucleic acid (DNA) damage, mitochondrial injury, and apoptotic cell death in the renal tubules. Furthermore, cisplatin reduces the bioavailability of nitric oxide, and it regulates monocyte chemoattractant protein-1, tissue growth factors, tumor necrosis factor-α, and reactive oxygen species [ROS]. cisplatin also causing kidney injury and inflammation [4]. In cells, cisplatin will interact with proteins and other cellular components such as microfilaments, cytoskeleton, and peptides containing thiol, RNA, and glutathione (GSH). The conjugation of cisplatin with GSH causes reactive thiols which are free radicals. Reactive thiols cause a decrease in vascular endothelial growth factor production, resulting in the disruption of the glomerular endothelial cell penetration [5]. Reactive thiols also trigger the death of proximal tubular cells due to oxidative stress so that antioxidants are needed to overcome them [6].

In Africa, Asia, Australia, and South America, *Coleus amboinicus* (CA) is known as a traditional plant with widespread growth; it is consumed daily for food supplementation or for treatment purposes [7]. CA plants have long been used by the community as a traditional herbal medicine for the treatment of various diseases such as heat, cough, bronchitis, sore throat, diarrhea, and dysentery and known to possess antimicrobial, antiepileptic, and antioxidant properties [8]. The coleus plant has 62 species that are divided into two groups (clade one and clade two) based on their DNA sequences. The active compounds in the CA plant species include monoterpenoid, sesquiterpenoid, diterpenoid, and phenolic [9], 3-methyl-4 isopropyl phenol, squalene, caryophyllene, phytol [10], alkaloids, glycosides, flavonoids, quinones, tannins, phenols, and terpenoids [11-13]. CA leaves have been used as a traditional food as a substitute for oregano as well as a usual ingredient in soup prepared to stimulate lactation [14].

This study aimed to investigate the activity of CA extract on the renal expression of transforming growth factor-1β (TGF-1β) in Wistar rats (*Rattus norvegicus*) with cisplatin-induced nephropathy.

## Materials and Methods

### Ethical approval

This research received ethical clearance and was registered with the Animal Care and Use Committee, Faculty of Veterinary Medicine, Airlangga University (Surabaya, Indonesia); registration number: 747-KE.

### Materials

This study had a true experimental design and included a randomized post-test control group. The CA plant was sourced from a traditional flower market in Surya Bratang, Surabaya, Indonesia. Fresh CA leaves were extracted using maceration with 96% ethanol. The CA extract was dissolved in a suspension containing 0.25% sodium carboxymethyl cellulose (CMC-Na). The CMC-Na suspension was used for treatment in the positive control group. The male Wistar rats (*R. norvegicus*) were purchased from Airlangga University, Animal Laboratory and were all between 2 and 3 months of age and weighed between 150 and 200 g. All rats were adapted with free access to food and tap water for a week in normal room temperature and under a 12 h light: dark cycle before the experiment for 7 days and were observed under clinical examination; no rats demonstrated any symptoms of illness.

All rats were divided into three groups, with eight animals per group; these included a control group (P0: normal saline), induction group (P1: received CMC-Na 0.25%), and a treated group (P2) that received daily orally of CA extract (500 mg/kg) for 7 days. Both P1 and P2 groups were induced with an intraperitoneal injection of cisplatin (Sigma-Aldrich, St. Louis, Missouri, USA) at a single dose of 5 mg/kg on the 4^th^ day of the experiment.

### Methods

All rats were sacrificed using anesthesia on the 8^th^ day for the collection of blood samples and kidney tissue. Kidney tissue was fixed in 10% buffered neutral formalin for histopathological and immunohistochemical (IHC) processing and analysis. Blood samples were collected for standard kidney function tests for assessing the concentrations of BUN, creatinine, and alkaline phosphatase using the colorimetric method (creatinine and BUN kit, DiaSys Diagnostic Systems GmbH, Holzheim, Germany) by measuring absorbance values in the samples after 1 min and comparing these with the standard solution. Expression of TGF-1β was identified using indirect immunoperoxidase monoclonal antibodies (mAB), secondary antiperoxidase antibodies, and by staining diaminobenzidine substrate with IHC kits (BD, Pharmingen) and labeled streptavidin-biotin (LSAB) from Starr Trek Universal Horseradish Peroxidase Detection System (Biocare, USA).

### Statistical analysis

Data obtained from histopathological and immunohistochemistry were analyzed on an Olympus Light Microscope CX 23 (PT. Fajar Mas Murni, Jakarta) by two pathologists. Kidney tissue sections were examined in areas containing immunoreactive glomerular cells under 100× and 400×. The aspects in evaluating the TGF-β1 expression included either a positive reaction (brownish aggregate) or a negative reaction (no color of brown aggregate) of immunostaining, color intensity, and distribution of immunopositive cells. The percentage of tissue area containing immunopositive cells was scored: Score 0 (if no alteration or the cells were normal), score 1 (has 1-30% positive cells), score 2 (has 31-50% positive cells), and score 3 (has 51-100% positive cells). All the statistical analyses were conducted using the Statistical Package for the Social Sciences for Windows, version 23.0 (IBM, USA). Values for the measured parameters were expressed as mean values ± standard deviation and the difference between the two groups was determined using an unpaired Student’s t-test. Histopathology and immunostaining scores were analyzed using non-parametric Kruskal–Wallis test and Mann–Whitney U-test and were expressed as mean values with a confidence interval of 95%. For all analyses, statistical significance was defined as p<0.05.

## Results

The mean BUN concentration for control rats was 51.83 mg/dL, which is within the considered normal range of 10-58 mg/dL. As shown in Table-1, the mean BUN concentration was 263.97 mg/dL in the P1 group, which was significantly greater than the BUN concentration in the P2 group (129.03 mg/dL; p<0.05). Creatinine levels in the control group (P0) were 0.68 mg/dL, which were within the normal range (0.20-0.80 mg/dL) [15]. Creatinine levels in Group P1 showed an average increase of 3.29 mg/dL, which was a significantly greater increase compared to P0 (p<0.05). Creatinine levels in Group P1 were significantly different from P2 group by 1.93 mg/dL.

**Table 1:** Comparison of concentrations in BUN, creatinine, and alkaline phosphatase in the control and treatment groups.

Group	BUN (mg/dL)	Creatinine (mg/dL)	Alkaline phosphatase (U/I)
P0 (n=8)	51.83±13.07	0.68±0.30	185.63±33.928
P1 (n=8)	263.97*±32.64	3.29*±0.73	531.38*±29.636
P2 (n=8)	129.03*±32.03	1.93*±0.41	274.63*±46.998

Superscript in the same column indicates a significant difference of p *≤*0.05. BUN=Blood urea nitrogen, n=Number of animals, P0=Negative control group, P1=Positive control group, P2=Treatment group

The concentrations of BUN, creatinine, and alkaline phosphatase were significantly (p≤0.05) increased in the induction group (P1) compared to the control group (P0).

**Figure-1:**
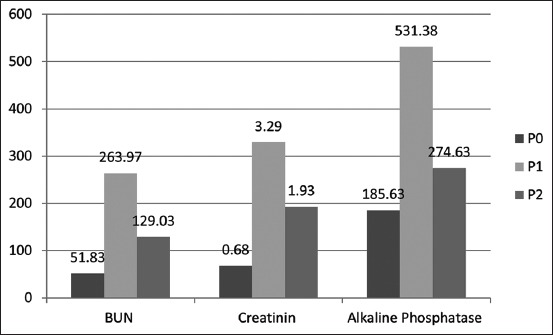
Effects of oral administration of *Coleus amboinicus* extracts on hematological parameters. The experimental results of the different treatments were analyzed using analysis of variance.

Effects of oral administration of *Coleus amboinicus* extracts on hematological parameters. The experimental results of the different treatments were analyzed using analysis of variance (Figure-1).

As shown in Table-2, the histopathology scores for the P1 group (which received CMC-Na) were 3.00 for degeneration and necrosis and 2.25 for the infiltration of cells. These scores were significantly greater (p≤0.05) than the necrosis and infiltration of cells scores (1.13 for both) for the P2 groups. The scores for the expression of TGF-1β were significantly different (p≤0.05) between treatment groups, with scores of 2.38, 3.00, and 7.88 in the P0, P1, and P2 groups, respectively.

**Table 2:** Histopathological scoring of degeneration, necrosis, infiltration, and TGF-1b immunoreaction in the control and treatment groups.

Group	Degeneration	Necrosis	Infiltration cell	TGF-1β
P0 (n=8)	0.25±0.46	0.00±0.00	0.00±0.00	2.38±0.74
P1 (n=8)	3.00*±0.00	3.00*±0.00	2.25*±0.88	3.00*±0.53
P2 (n=8)	3.00*±0.00	1.13*±0.35	1.13*±0.35	7.88*±1.55

Superscript in the same column indicates a significant difference of p ≤0.05. TGF-1β=Transforming growth factor-1β, n=Number of animals, P0=Control group, P1=Induction group, P2=Treated group

Nephropathy is characterized by a high BUN concentration, which occurs due to the hypovolemia and resulting dehydration in the kidney tubules [16,17]. The standard test for determining renal function status is by measuring serum creatinine and urine production, but serum creatinine measurements may not always reflect kidney injury [18,19]. The previous studies have shown that creatinine levels in rats increase after cisplatin induction. This is due to a decrease in kidney function caused the effect of cisplatin [20].

**Figure-2:**
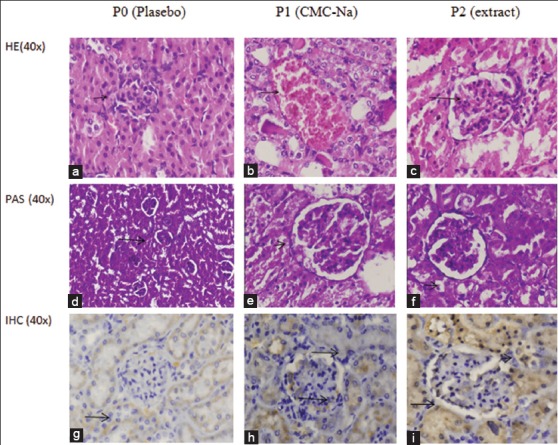
(a and d) The control group’s histopathology showed normal glomerular and tubular cell structures (hematoxylin and eosin/periodic acid–Schiff [HE/PAS], 40×). (b and e) Histopathology Group P1 shows severe hemorrhagic and necrotic cell in the area of the proximal convolutes tubules and thickening of the contortus tubule basement membrane and capsule Bowman in the renal glomerulus (HE/PAS, 40×). (c and f) Treatment group of extract showed vacuolar degeneration alteration (HE/PAS, 40×). (Change is indicated by arrows). (g) The control group labeled with transforming growth factor-1β (TGF-1β) monoclonal antibody (mAb) showed no positive reaction. (h) In the induction P1 group, there was a positive specific reaction to TGF-1β antibodies which were marked with a golden-brown color on the tubular membrane epithelium and glomerular mesangial cell. (i) The picture in ethanolic coleus extract P2 group demonstrates a positive reaction specific to mAb. TGF-1β showed a more homogeneous brownish aggregate in the area of epithelial tubules and glomerulus (immunohistochemistry, 40×) (change shown by arrow).

Histopathological changes showed a failure in kidney function, as evidenced by severe hemorrhagic, inflammation, and necrosis in the areas of renal tubules and interstitium. Furthermore, the thickening of capsule Bowman in the areas of the renal glomeruli was evidenced in animals induced with cisplatin (Figure-2). Meanwhile, the staining of kidney tissue labeled with mAB to TGF-1β showed an increase in expression of TGF-1β, which was characterized by a brown aggregate on the epithelial surface of the tubules and on mesangial cells in the glomeruli in the P1 and P2 treatment groups.

## Discussion

The results of induction with cisplatin administration in the treatment groups P1 and P2 showed that all treatment groups experienced impaired kidney function, which was marked by an increase in BUN, creatinine, and alkaline phosphatase levels. A recent study suggested that acute kidney injury is the most serious side effect of cisplatin, an important antitumor agent. Lipid peroxidation and oxygen free radicals are important mediators of cisplatin-induced nephrotoxicity [21]. The molecular role of TGF-1β is critical in driving mechanisms of cellular repair. However, the repair process is not beneficial to all tissues as TGF-1β also mediates tissue fibrosis. In Wistar rats induced with uric acid, the increase in TGF-1β concentrations is an indicator of decreased necrotic tissue damage and of the kidney tissue being repaired. The previous studies showed that the expression of TGF-1β in tubular cells plays a role in tubulointerstitial fibrosis [22,23]. Furthermore, TGF-1β expression plays a role in regulating mesangial cell proliferation and extracellular matrix (ECM) secretion in diabetic nephropathy. *In vivo* expression of TGF-1β influences collagen gene expression and mesangial ECM synthesis in the glomerular mesangium. Accumulation of ECM can cause tubulointerstitial fibrosis, thickening of the glomerular basement membrane, and glomerular sclerosis [24,25]. However, TGF-1β expression could induce profibrogenic mechanism, independently through the activation of epidermal growth factor receptors and activation of angiotensin II, endothelin-1, and oxidative stress conditions involved in the renal fibrogenesis processes. In rats induced with cisplatin, the increased expression of TGF-1β indicates tissue fibrosis in the glomerulus and renal tubules and is one of the causes of kidney failure in these animals [26,27]. The mechanism of cisplatin-induced nephrotoxicity is complex and involves the accumulation of cisplatin membrane transportation, DNA damage, mitochondrial dysfunction, and oxidative stress and inflammatory response. ROS production, depletion of antioxidant systems, and stimulation of renal accumulation of lipid peroxidation products have all been suggested as key mechanisms contributing to cisplatin-induced nephrotoxicity [28]. The present study showed that the administration of CA extract (P2) increased TGF-1β expression in the area of renal tubules and glomerulus. Furthermore, alteration in the renal tubules and glomerulus showed reversible injury by vacuolation in the cytoplasm and degeneration lesions in the treatment group with CA had distinction with CMC-Na (P1). According to Castillo-Sánchez *et al*. (2018), the secondary metabolites of CA allow it to present antioxidant, antibacterial, antitumor, cicatrizing, antiepileptic, larvicidal, anti-inflammatory, analgesic, insecticidal, repellent, and acaricide activity [29]. A previous study conducted by Mansour and Ghobara (2015) showed that the administration of cisplatin-induced various degenerative changes in kidney cells, confirming the biochemical evidence of the oxidative stress, which had been previously mitigated by the pre-treatment of herbal extract [30]. Renal tubule necrosis is cell death due to injury in a host. In necrotic cells, there is an increased density of chromatin in the nucleus, which becomes wrinkled, appears denser, has a black coloration (piknosis) and is fragmented (kariolysis) [31,32]. This means that the administration of CA extract in Wistar rats might reduce cisplatin-induced renal cell damage and indicate that this extract exerts an inhibitory effect on the process of kidney fibrosis. CA extract could prevent the side effects of cisplatin induction. In another study that assessed the effects of aqueous leaf extract, CA demonstrated significant nephroprotective effects at the equivalent therapeutic dose of 400 mg/kg against adriamycin-induced acute nephrotoxicity [14,33]. Notably, in the control group in this study, CA treatment showed no alterations in renal tissue histopathology, indicating that the kidneys were in normal condition.

## Conclusion

The present study showed that CA might regulate the increase of TGF-1β expression in areas of the kidney containing the glomeruli and tubule interstitium. It is considered that histopathology lesions could be related to acute renal injury by cisplatin-induced nephropathy in Wistar rats and the inhibition of renal necrosis and infiltration of cells might be influenced by TGF-β1.

## Authors’ Contributions

RS designed and performed the study. IS collected the samples and analyzed the data. RS wrote the first draft of the manuscript. IS revised the manuscripts. All authors read and approved the final draft of the manuscript.
